# Brachytherapy boost in loco-regionally advanced nasopharyngeal carcinoma: a prospective randomized trial of the International Atomic Energy Agency

**DOI:** 10.1186/1748-717X-9-67

**Published:** 2014-03-01

**Authors:** Eduardo Rosenblatt, May Abdel-Wahab, Mahmoud El-Gantiry, Inas Elattar, Jean Marc Bourque, M’hamed Afiane, Nouredine Benjaafar, Shahid Abubaker, Yaowalak Chansilpa, Bhadrasain Vikram, Peter Levendag

**Affiliations:** 1 International Atomic Energy Agency, Vienna, Austria; 2 Taussig Comprehensive Cancer Center, Cleveland Clinic, Cleveland, OH, USA; 3 National Cancer Institute, University of Cairo, Cairo, Egypt; 4 Départment de Radiothérapie, Centre Pierre et Marie Curie, Centre Hospitalier Universitaire Mustafa (CHU), Alger, Algeria; 5 Institut National d'Oncologie, Rabat, Morocco; 6 Institute of Nuclear Medicine and Oncology, Pakistan Atomic Energy Commission (PAEC), Lahore, Pakistan; 7 Siriraj Hospital, Faculty of Medicine, Mahidol University, Bangkok, Thailand; 8 National Cancer Institute, Bethesda, MD, USA; 9 Erasmus University, Rotterdam, Netherlands; 10 London Regional Cancer Program, University of Western Ontario, London, Canada

**Keywords:** Nasopharynx, Nasopharyngeal carcinoma, Brachytherapy boost

## Abstract

**Abstact:**

**Background**

The purpose was to determine whether a brachytherapy boost improves outcomes in patients with advanced nasopharyngeal carcinoma treated with standard chemo-radiotherapy.

**Methods**

Patients with nasopharyngeal carcinoma WHO grades I-III and TNM stages III or non-metastatic stage IV were eligible for this phase III study. Patients were randomized to either arm (A) induction chemotherapy, followed by external beam radiotherapy (EBRT) with concomitant cisplatin (n = 139) or arm (B), the same schedule plus a brachytherapy boost to the nasopharynx (n = 135). The EBRT doses given were 70 Gy to the primary tumour and positive lymph nodes and 46 Gy to the negative neck. The additional brachytherapy boost in arm (B) was given by either low dose-rate (LDR – 11 Gy) or high dose-rate (HDR – 3 fractions of 3.0 Gy) brachytherapy. The primary endpoint was 3-year overall survival (OS) and secondary endpoints were: local control, regional control, distant metastasis and grade 3–4 adverse events.

**Results**

274 patients were randomized between September 2004 and December 2008. The two arms were comparable with regard to age, gender, stage and grade. 273 patients completed treatment. Median follow-up was 29 months (0.2-67 months). The effect of treatment arm, country, age, gender, WHO pathology, stage (T3-4, N2-3 versus other) and chemotherapy on overall survival (OS), disease-free survival (DFS) and local recurrence-free survival (LRFS) was studied. Stage significantly affected OS (p = 0.024) and DFS (p = 0.018) while age significantly affected OS (p = 0.014). None of the other factors studied were significant. The 3-year LRFS was 60.5% and 54.4% in arms A and B respectively (p = 0.647). The 3-year regional control rate in the neck was 59.7% and 54.3% respectively (p = 0.7). Distant metastasis developed in 59.7% of patients in arm A and 55.4% in arm B (p = 0.377). Patients with T1/T2 N + had a 3 year LRFS of 51.8% in Arm A (62 patients) versus 57.9% in Arm B (67 patients) (p = 0.343). The grade 3–4 toxicity rate was 21.6% (30/139) and 24.4% (33/135) respectively (p = 0.687).

**Conclusions**

The addition of a brachytherapy boost to external beam radiotherapy and chemotherapy did not improve outcome in loco-regionally advanced nasopharyngeal carcinoma.

## Background

Nasopharyngeal carcinoma (NPC) is among the cancers which are common and potentially curable with radiotherapy in Asia and North Africa. However, patients treated with radiotherapy alone have a cumulative incidence of persistent local disease of up to 13% and a 10-year actuarial local failure-free survival of 61% [1]. Thus, local recurrences represent a significant component of radiation failures. In addition to local symptoms related to local recurrence, lack of local control is an independent prognostic factor for the development of distant metastases. Local control may be related to the total dose of radiation given through external beam radiotherapy (EBRT) alone or combined with brachytherapy. Previous studies report an acceptable incidence of side effects with cumulative doses of up to 81 Gy to the nasopharynx proper [2-4]. Neoadjuvant chemotherapy was found to improve outcomes in randomized studies. Al-Sarraf et al. [5] reported a significant improvement in 3-year survival (78% vs. 47%) as well as reduction in local and distant failure rates in the arm that received concomitant cisplatin and adjuvant chemotherapy.

The present study was designed to determine the potential clinical benefit of radiotherapy dose escalation using brachytherapy, in addition to the standard external beam radiation and chemotherapy commonly used in the treatment of nasopharyngeal carcinoma, in patients with locally- or loco-regionally advanced disease.

The present study was a multicentre, international clinical trial coordinated by the International Atomic Energy Agency (IAEA), with data management and statistics conducted at the National Cancer Institute of Cairo, Egypt and patient accrual and treatment implemented in radiotherapy centres in Egypt, Algeria, Morocco, Pakistan and Thailand.

## Patients and methods

The protocol was approved by the Institutional Review Board of the participating hospitals and all patients provided informed consent before trial entry. Adult patients with histopathologically proven nasopharyngeal carcinoma (WHO type I-III) and T3-T4 N0-3 or T1-T2, N2, N3 disease, according to the TNM classification of the Union for International Cancer Control (UICC), 5th edition, were eligible for inclusion in the study. Patients had to be over 15 years of age and have an Eastern Cooperative Oncology Group (ECOG) performance status of 0–2 to be eligible. Laboratory tests had to be within normal range and patients with inadequate haematological, renal or hepatic function were excluded (i.e.: Hgb <10 g/dl, WBC < 4000, absolute neutrophil count (ANC) <1500, platelets < 100,000, serum bilirubin >1.5 mg/dl, serum creatinine >1.5 mg/dl). Patients were ineligible for trial participation if they were pregnant or lactating, if they had metastatic (M1) disease, if they were previously treated for their cancer, or had a simultaneous or prior malignancy with the exception of non-melanoma skin cancers or carcinoma in-situ of the cervix.

Work-up of study patients included the following laboratory tests: complete blood count (CBC), liver function tests and renal function tests (BUN, serum creatinine/creatinine clearance). Imaging included chest x-ray, head and neck CT-scan and abdominal ultrasound. Cardiac function was evaluated by ECG and left ventricular ejection fraction. Bone scan/skeletal survey and serum alkaline phosphatase were used to assess for bone metastases. Bone scan was mandatory in case of bone symptoms or elevated serum alkaline phosphatase. Pre-treatment oral/dental consultation was required for all patients.

The standard arm included neoadjuvant chemotherapy and concurrent chemo-radiation therapy, consisting of EBRT alone. Treatment in the study arm consisted of a similar regimen of neoadjuvant chemotherapy, concurrent chemo-radiation therapy with EBRT plus the addition of a brachytherapy boost.

Neoadjuvant chemotherapy consisted of cisplatin: 100 mg/m^2^ and doxorubicin 50 mg/m^2^ or Epirubicin 75 mg/m^2^, with hydration, diuretics and antiemetic drugs. Neoadjuvant chemotherapy was given every 3 weeks for 2 cycles. The first course of neoadjuvant chemotherapy was given on day 1 and the second course on day 22. Each course was postponed for 1 week if the CBC was not acceptable for treatment (WBC < 3000; ANC < 1500; Platelet < 80 000). Chemotherapy was discontinued if there was no improvement in CBC, if intercurrent disease occurred, if creatinine was >1.5 times the normal level or if the patient refused further chemotherapy. Chemotherapy was also discontinued if the patient developed cardiac symptoms and/or ECG and echocardiography were to be done to monitor cardiac function.

Neoadjuvant chemotherapy was followed by EBRT starting between day 43 to day 50 and concurrent weekly cisplatin 30 mg/m2 /week throughout the radiotherapy course (7 weeks). Radiation therapy was scheduled to start within 6 hours of the cisplatin infusion. Subsequent to the completion of the external beam radiation, only patients randomized to the experimental arm received a brachytherapy boost. Per protocol, brachytherapy was to be given within 1 week of the completion of EBRT.

Concomitant chemotherapy could be stopped for the same reasons as neoadjuvant chemotherapy. Radiation therapy could be delayed for up to one week if grade 3–4 hematologic toxicities were seen.

Megavoltage machines were used (Co-60 or 4–6 MV linear accelerators) with mask immobilization in an extended neck position. The treatment fields, shielding blocks and entrance point of each field were marked on the mask. Nodal masses in the neck were marked with metallic wire. Simulator films were taken. The total tumour dose was 70 Gy in 35 fractions (2.0 Gy per fraction) given over 7 weeks.

The initial treatment volume included the primary tumour and its extensions, posterior and upper deep cervical lymph nodes which were treated through two parallel opposed lateral fields. Setting the field junction over enlarged lymph nodes was avoided when possible. The clinical target volume included the nasopharynx, the skull base, the sphenoidal and posterior ethmoid sinuses, the floor of the middle cranial fossa, the posterior half of the nasal cavity and orbit, the lateral pharyngeal walls and the parapharyngeal space, inferior extension to the oropharynx, posterior cervical, deep cervical and supraclavicular lymph nodes. The lower border of the lateral fields was placed high in the neck to avoid the unnecessary inclusion of the larynx. The optic chiasm, brainstem, anterior half of the orbit and a portion of the oral mucosa were shielded if there was no tumour extension. Patients with anterior nasal extension were treated with an additional anterior photon field. Mid and low cervical nodes were treated through a direct anterior field. If there were very large cervical lymph nodes; two large lateral opposed fields extending from skull base to the clavicles were used to avoid placing a field junction over the area of lymph node disease whenever possible. Reassessment of the lymph node size was carried out at 20–40 Gy. The treatment fields were then modified into two parallel opposed lateral fields to the upper head and neck and a direct anterior lower neck field as long as the lymph node size reduction was sufficient to avoid field junction over the lymph nodes. For the initial fields, the dose to the primary tumour was 40 Gy/20 fractions/4 weeks (2 Gy /fraction). Regional neck lymph nodes received doses based on lymph node stage: N0 dose was 46 Gy at 3 cm depth, with an off cord field modification after 40 Gy to allow for an electron field over the posterior neck to deliver 6 Gy /2-3 fractions /2-3 days. For the boost portion of the treatment with EBRT, the primary tumour site and extensions was treated with two lateral opposed reduced fields. The whole lower neck was treated through a large direct anterior field with shielding of the spinal cord. The posterior spinal lymph nodes were treated with electron beam (> 9 MeV). Optic chiasm, optic nerves and cervical spinal cord limits of not more than 45 Gy were allowed. The doses delivered through the boost field were as follows: primary tumour: 30 Gy/15 fractions/3 weeks; regional lymph nodes: N1-3: 30 Gy. Therefore, the final total dose to the primary tumour and clinically involved lymph nodes was 70 Gy and 46 Gy to the clinically negative neck.

**Figure 1:**
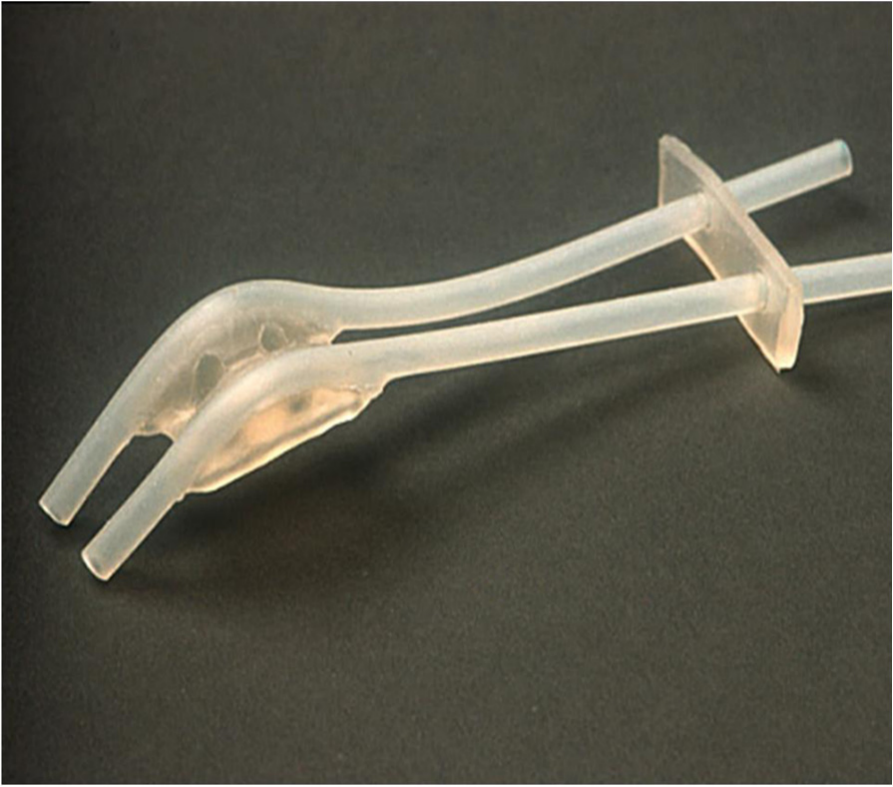
The Rotterdam nasopharyngeal applicator for brachytherapy.

The silicone Rotterdam Nasopharynx Applicator [6] (Figure 1) was used to deliver brachytherapy in the experimental study arm. Investigators were specifically trained in the use, insertion, imaging and dosimetry of the Rotterdam applicator which was used in all cases since this applicator allows for either low-dose-rate (LDR) or high-dose-rate (HDR) brachytherapy.

**Figure 2:**
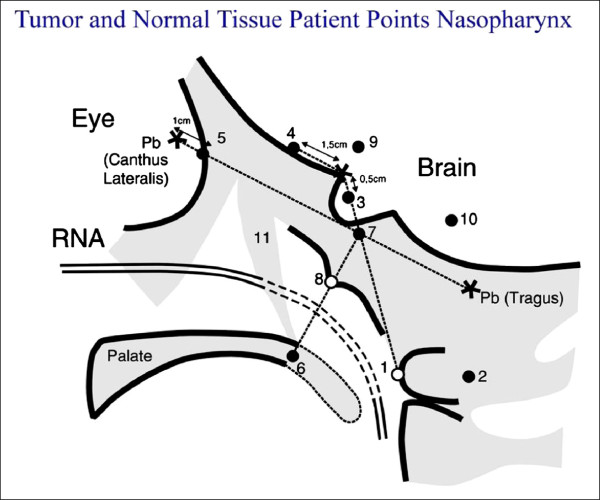
Dosimetry points for the Rotterdam nasopharyngeal applicator in the lateral view: 1 Node of Rouvière point, 2 Spinal cord point, 3 Pituitary gland, 4 Optic chiasm, 5 Retina, 6 Soft palate, 7 Base of skull, 8 Nasopharynx, 9 Temporal lobe, 10 Inner ear, 11 Pterygoid plates.

The applicator was introduced transorally under topical anaesthesia, and fixed in position in the nasal cavity and nasopharynx. Then, standard afterloading catheters were inserted into the applicator’s silicone tubes. In case of LDR, two Ir-192 wires with an average activity of 50–60 mCi/cm, were inserted into both afterloading catheters. For HDR, the afterloading catheters were connected to a remote-controlled afterloader (Ir-192 microSelectron HDR). The applicator remained in place for the duration of the treatment for both LDR- and HDR endocavitary irradiation. In case of LDR, the active source was tailored to the nasopharynx proper that is to the distance between the ‘Node of Rouviere’ (at the level of the C-I vertebral body), [6] (Figure 2) and the pterygoid plates. The Ir-192 source was removed after a dose of 11 Gy was delivered to the nasopharynx “tumour tissue” (TT) point. For HDR, a boost dose of 9.0 Gy in 3 fractions of 3.0 Gy each was delivered with a minimum interval of 6 hours between fractions. The dose was prescribed to the “tumour tissue” (TT) points ‘Node of Rouviere (R)’ and/or ‘Nasopharynx (Na)’. The dose distribution for LDR and HDR was computed in the ‘Tumour Tissue (TT)’ points ‘Na’ and ‘R’, as well as in the critical normal tissue (NT) points representing the soft palate, base of skull, pituitary gland, optic chiasm, and retina. Other optional points representing normal tissue were the temporal lobes, and inner ear. These dosimetry points have been previously described by Levendag et al. [6].

In the study arm, both low dose-rate (LDR) and high dose-rate (HDR) brachytherapy were allowed for the nasopharynx boost. The brachytherapy dose schedules were: for LDR: 11 Gy to the “nasopharyngeal tumour tissue point” and for HDR: 3 fractions of 3.0 Gy each (total: 9.0 Gy) to the same point, with a minimum interval of 6 hours between fractions. RTOG (Radiation Therapy Oncology Group) acute radiation morbidity scoring criteria and RTOG/EORTC (European Organization for the Research on the Treatment of Cancer) late radiation morbidity scoring criteria were used to assess toxicity.

### Statistics

Stratification according to treating centre and stage (T4N3 vs. others) was done. The primary endpoint was overall survival at 3 years. It was hypothesized that the survival rate would be 65% (or greater) in the experimental arm versus 50% in the control arm. Secondary endpoints included: response rate at 40 Gy, local control rate (local relapse-free survival), rate of distant metastases (distant metastases free survival) and toxicity rate. Response rate at 40 Gy was not evaluated in the final analysis due to insufficient data. Patients were randomized to two arms, a standard/control and a study arm. A sample size calculation was done to determine the number of patients needed in each arm to allow a comparison of 50% survival rate with the control treatment, to the study arm with an hypothesized survival rate of 65%, for a test of significance level of 0.05 and 80% power. Interim analyses were planned to allow early termination of the study if the number of treatment-related deaths in the experimental arm exceeded 4 or if the absolute difference in survival between the two arms exceeded 20%.

## Results

Between September 2004 and December 2008 a total of 274 eligible patients were centrally randomized to either the standard arm (arm A) which consisted of 2 courses of induction chemotherapy, followed by external beam radiotherapy with concomitant cisplatin (n = 139) or the study arm (arm B) which consisted of the same schedule plus an additional brachytherapy boost to the nasopharynx using the Rotterdam applicator (n = 135). One patient who was initially randomized to the study arm was immediately lost to follow up and had no outcome data; therefore, was not included in the analyses. The distribution of stage, gender and histological grades were statistically comparable in both treatment arms (Table 1). However, patients in the study arm were younger by approximately 3.5 years (p = 0.044). The median follow-up was 29 months (2–67 months) and 9% patients were lost to follow-up. The patients received treatment as follows:

**Table 1:** Patients’ demographics in the two study arms

		**Standard**	**Brachytherapy**	
		**n = 139**	**n = 135**	
**Measurement**		**n (%)**	**n (%)**	P-value**
Age (years)*		43.5 ± 13.6	40.0 ± 14.8	0.044
Gender	Male	104 (74.8)	94 (69.6)	
	Female	35 (25.2)	41 (30.4)	0.337
WHO histology	1-2	29 (20.9)	37 (27.4)	
	3	110 (79.1)	98 (72.6)	0.205
Stage	T3-4&N2-3	34 (24.5)	36 (26.7)	
	Others	105 (75.5)	99 (73.3)	0.676
Neoadjuvant chemo	Yes	120 (86.3)	119 (88.1)	
	No	19 (13.7)	16 (11.9)	0.652

*Value is mean ± standard deviation. **P-values ≤ 0.05 are considered significant.

P-values ≤ 0.05 are considered significant.

Two hundred and seventy-five patients were found eligible and randomized. Age, gender and the distribution of stages and histological grades were statistically comparable in both treatment arms. 94% of patients completed the treatment as planned. The median follow-up was 29 months (2–67 months) and 9% patients were lost to follow-up.

External beam radiation: 257 patients received 70 Gy in 35 2.0 Gy fractions with EBRT. Seven patients had missing dose information. An additional eight patients received the following lower doses of radiation: 10 Gy (1 patient), 30 Gy (1 patient), 40 Gy (4 patients), 56 Gy (1 patient), 57 Gy (1 patient). Also, there was one additional patient who refused external beam radiation in the standard arm. Thus, of 273 patients 272 had external beam radiation therapy, 94% of them to the prescribed protocol dose.

Out of the 135 patients who were randomized to the experimental arm, one hundred and seven patients did receive the brachytherapy treatment (80%), twenty five patients did not, and data was missing on two patients.

Patients had the brachytherapy procedure done a median of 10 days after the external beam radiation ended (range 0–52 days). Of the remaining patients who received brachytherapy implants, 30 (22%) had the procedure within a week of completing the EBRT. Brachytherapy implants were done at 2, 4 and 6 weeks or over 6 weeks in the remaining 39, 22, 10 and 3 patients, respectively.

One hundred and forty nine patients received 70 Gy in 35 fractions. One hundred and eight patients received doses somewhat higher than 70 Gy (79 Gy and 81 Gy in 61 and 47 patients respectively). The cumulative (combined external beam and brachytherapy) radiation doses in the remaining patients were as follows: 10 Gy (1 patient), 30 Gy (1 patient), 40 Gy (2 patients), 51 Gy (2 patients), 56 Gy (1 patient), 57 Gy (1 patient). One patient randomized to the standard arm, subsequently refused treatment.

Cisplatin concomitant chemotherapy was used in all patients, with the exception of 3 patients who received no concomitant chemotherapy, two in the standard arm and one in the study arm.

Information about the number of cycles was available in most patients. Twenty three of 139 patients (17%) in the standard arm did not receive neoadjuvant chemotherapy. In the brachytherapy arm, 16 of the 135 patients (12%) did not receive neoadjuvant chemotherapy. In the standard arm, the number of chemotherapy cycles given was 2 cycles in 111 patients (23 received cisplatin/5FU; 88 received cisplatin/doxorubicin) and 1 cycle in two patients (one of each chemotherapy regimen). In the brachytherapy arm, the number of chemotherapy cycles given was 2 cycles in 115 patients (85 received cisplatin/doxorubicin and 30 received cisplatin/5FU).

Although not part of the initial study endpoints, a comparative analysis between induction chemotherapy with cisplatin/doxorubicin (n = 155, 57%) and patients who received cisplatin/5FU (n = 118, 43%) showed a 3-year DFS rate of 62.3% for the former and 53.9% for the latter (p = 0.001) indicating that induction treatment with cisplatin/5FU was inferior. A multivariate analysis of prognostic factors showed that only TNM stage and chemotherapy with cisplatin/5FU (118 patients -43%- treated in Algeria) were adverse prognostic factors for survival in this series.

One hundred and fifty-five patients were to receive cisplatin/doxorubicin, while 118 patients at one specific centre received cisplatin/5FU (Table 2). When patients receiving cisplatin/5FU are compared to patients who received cisplatin/doxorubicin, no significant difference was seen in age (p = 0.739), gender (p = 0.109), WHO histology (p = 0.174), but there was a significant difference observed in stage (p = 0.034).

**Table 2:** Patient characteristics in the two groups that received different neoadjuvant chemotherapy with Cisplatin and Doxorubicin and patients from Algeria who received Cisplatin and 5 FU

		**Algeria**	**Other countries**	
		**n = 119**	**n = 155**	
**Measurement**		**n (%)**	**n (%)**	**P-value**
Age (years)*		42.8 ± 13.3	41.0 ± 15.0	0.303
**Gender**	Male	82 (68.9)	116 (74.8)	
	Female	37 (31.1)	39 (25.2)	0.277
WHO histology	1-2	24 (20.2)	42 (27.1)	
	3	95 (79.8)	113 (72.9)	0.184
Stage	T3-4&N2-3	27 (22.7)	43 (27.7)	
	Others	92 (77.3)	112 (72.3)	0.342

*Value is mean ± standard deriation.

P-values ≤ 0.05 are considered significant.

### Results by study arm

**Figure 3:**
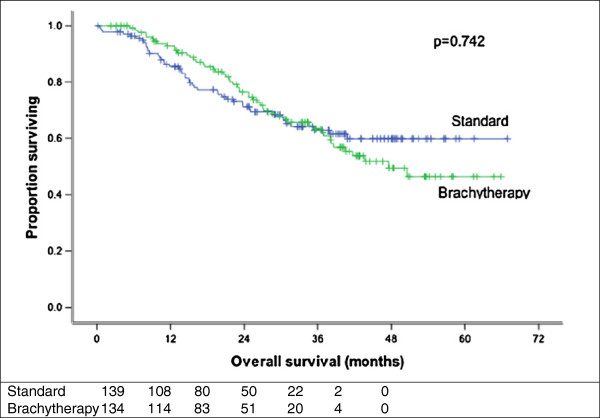
**Overall survival by randomization arm.** The difference is not statistically significant (p = 0.742).

**Figure 4:**
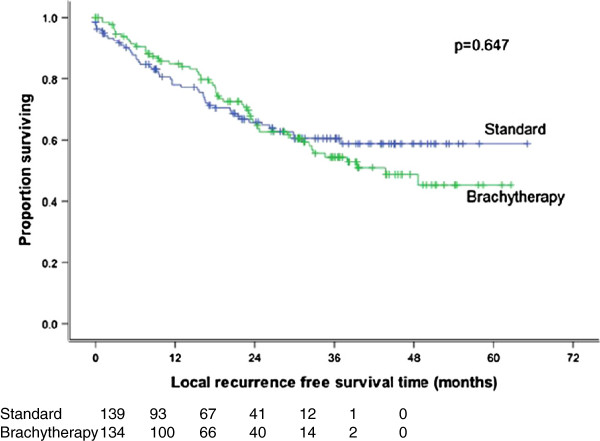
**Local recurrence free survival by randomization group.** No statistically significant difference.

Overall survival, local relapse free and disease free survival were not significantly different between treatment arms (Figures 3 and 4). The 3-year overall survival rate was 62.9% and 63.3% (p = 0.742) (Figure 3) for standard and study arms, respectively; while the 3-year disease-free survival was 59.8% and 52.6% (p = 0.496) (Table 3).

**Table 3:** Patient’s characteristics and outcomes: overall survival, disease-free survival and local-recurrence free survival

**Measurement**		**Number of patients**	**3-year overall survival (%)**	P value*	**3-year disease free survival (%)**	P value*	**3-year local relapse free survival (%)**	P-value*
Treatment	Standard	139	62.9	0.742	59.8	0.496	60.5	0.647
	Brachytherapy	134	63.3		52.6		54.4	
Country	A	118	59.8	0.222	49.9	0.124	51.7	0.283
	B	29	45.2		37.2		40.9	
	C	76	71.3		67.1		67.1	
	D	25	70.1		60.0		60.0	
	E	25	68.8		68.7		68.7	
Age (years)	<40 yrs	105	71.5	0.014	64.4	0.125	66.8	0.058
	≥40 yrs	168	57.6		50.6		51.1	
Sex	Male	197	63.3	0.540	56.2	0.442	57.6	0.394
	Female	76	61.5		55.8		56.6	
WHO pathology	1-2	65	61.4	0.949	53.9	0.461	54.8	0.438
	3	208	63.6		56.7		58.0	
Stage	T3-4 & N2-3	70	50.6	0.024	45.3	0.018	46.2	0.016
	Others	203	66.5		59.3		60.6	
Chemotherapy	No	35	75.2	0.159	65.4	0.136	65.4	0.184
	Yes	238	60.4		54.7		56.2	
T Stage	T1&T2	140					59.0	0.372
	T3&T4	133					55.3	
T Stage & N	T1/T2N+	129					54.9	0.683
	Others	144					59.8	

*P-value ≤ 0.05 is considered significant. WHO = World Health Organization. N = Lymph node status.

The 3-year local recurrence free survival rate was 60.5% and 54.4% (p = 0.647) (Figure 4) for the standard and study arm, respectively. Moreover, the latter was not significantly different between treatment arms, even when stratified by different T and N stage combinations (e.g.: T1-T2 N + versus others) (Table 4). Fifty one percent of patients developed distant metastasis in arm A and 46% in arm B (p = 0.377). Distant metastasis-free survival (DMFS) was not significantly different by treatment arm (p = 0.43).

**Table 4:** Three-year survival by treatment arm stratified by T1-T2 N + stage

**T – N stage**	**Measurement**	**Number of cases**	**3 years survival (%)**	P-value*
T1&T2N+	Standard	62	51.8	
	Brachytherapy	67	57.9	0.343
Others	Standard	77	67.9	
	Brachytherapy	67	50.0	0.106
T1&T2	Standard	70	58.2	
	Brachytherapy	70	60.1	0.641
T3&T4	Standard	69	63.2	
	Brachytherapy	64	46.3	0.205

*P-value ≤ 0.05 is considered significant.

Regional relapse free survival (RRFS) was not significantly different by treatment arm (p = 0.70). The only factors affecting regional relapse free survival were age <40 years versus > 40 years (66.9% 50.6%; p = 0.047) and advanced stage T3-4, N2-3 vs. others (45.4% vs. 60.2%; p = 0.023).

Analysis by actual treatment received (i.e. EBRT alone versus EBRT and brachytherapy), rather than intention-to-treat, also showed that brachytherapy did not significantly affect local control (p = 0.906) or overall survival (p = 0.819).

Significant variables by Log rank test were entered into a Cox Proportional Hazards model to study the independent effect of the prognostic factors on all survival estimates. Significant variables were stage (T3 or T4 and N2 or N3 versus other stages) as well as age (<40 yrs vs ≥40 yrs). Although the treatment arms used were not a significant factor, they were also entered into the Cox model. Stage and age were still the only significant factors found after performing multivariate analyses. The odds of death among patients with stage T3 or T4 and N2 or N3 was 1.77 higher than the other stages, with a (95% CI: 1.14-2.74) while the odds of death was 1.81 (95% CI: 1.17-2.80) higher among old age groups compared to the younger age group.

Grade 3–4 toxicity rate was not significantly different between study arms (Table 5).

**Table 5:** Late treatment toxicities

	**Arm 1 (No BT)**		**Arm 2 (BT)**		**P-value**
	**GI-II**	**GIII-IV**	**GI-II**	**GIII-IV**	
Skin	137	2	130	5	0.277
Subcutaneous tissue	136	3	132	3	1.000
Mucous membranes	138	1	131	4	0.209
Salivary glands	131	8	126	9	0.806
Brain	138	1	135	0	1.000
Trismus	124	15	123	12	0.687

## Discussion

### Rationale of using brachytherapy in the advanced-disease setting

Modern radiotherapy techniques such as 3D conformal radiotherapy, IMRT and stereotactic radiotherapy are the mainstay of treatment of nasopharyngeal carcinoma with very encouraging outcomes [7-11]. In low- and lower-middle income countries however, many radiotherapy centres still rely on a 2D technique to treat the numerous patients referred to them. Until these centres adopt 3D-CRT as a standard, they will need to optimize the resources available.

Several retrospective analyses have addressed the role of a brachytherapy boost in nasopharyngeal carcinoma [12-18]. These studies have looked at brachytherapy as a technique to escalate the dose to the primary tumour and extensions without incurring in unacceptable toxicity to surrounding sensitive organs. To our knowledge, this is the first prospective randomized study of dose escalation using a brachytherapy boost after external beam radiation therapy in patients with advanced local or loco-regional disease.

Patients with advanced loco-regional disease were specifically targeted for this study for two reasons: (1) they represent the majority of patients with NPC seen in low-middle income countries where the disease is common, and (2) the brachytherapy boost was applied following full high-dose external beam radiotherapy with neoadjuvant as well as concomitant chemotherapy. Therefore, the intention was to treat the residual disease after the first phase of intensive treatment.

Of the published retrospective studies, some are limited by small patient numbers and differing stages and pathology. In spite of these study limitations, several authors have reported improvement in outcomes with dose-escalation using brachytherapy in conjunction to the standard external beam radiation. In 1980, Chang et al. [19] was the first to report an improvement in survival with the addition of intracavitary radium brachytherapy to Cobalt-60 teletherapy (68.6% vs. 40.4%, p < 0.01). Furthermore, in a retrospective review of 509 patients, Teo et al. [16] reported an increase in local control when additional HDR brachytherapy was used after external beam radiation therapy. This benefit of brachytherapy was not consistently reported, however. A recent series from Haceteppe University [17] did not show any improvement in local control in patients treated with additional brachytherapy implant after external beam radiation as compared to external beam radiation alone (86% vs. 94%; p = 0.23). One explanation may be that the patients in the EBRT alone arm were significantly younger and more likely to receive chemotherapy which may have favourably affected their outcomes. Furthermore, patients received brachytherapy 4–6 weeks after EBRT which may have influenced the results due to a protracted overall treatment time. On the other hand, the longer interval may also allow time for a decrease in tumour size, thus enhancing the brachytherapy dosimetry. The results of the latter study concurred with the findings from the present study where no differences in outcome were seen when an additional brachytherapy boost was added to the external beam radiation. Analyses, in the present study, by both intention-to-treat and by actual treatment received, showed no differences between both arms with respect to 3 year overall survival (p = 0.819 and p = 0.742, respectively) and local relapse free survival (p = 0.906 and p = 0.496, respectively).

Patient characteristics were equally distributed between the two randomized groups with the exception of age where the brachytherapy patients were younger by approximately 3.5 years (p = 0.044). Thus patients in the study arm may have a relative advantage since older age is a worse prognostic factor. In spite of this, the addition of brachytherapy did not significantly improve patient’s outcome in this study.

**Figure 5:**
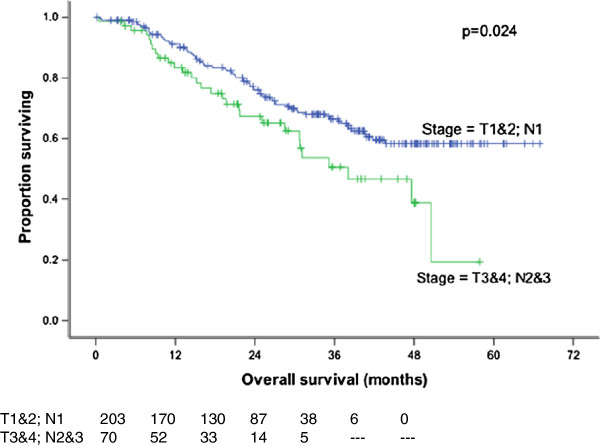
**Overall survival by stage.** TNM stage had a significant impact on overall survival which was favourable for patients with early disease (p = 0.024).

**Figure 6:**
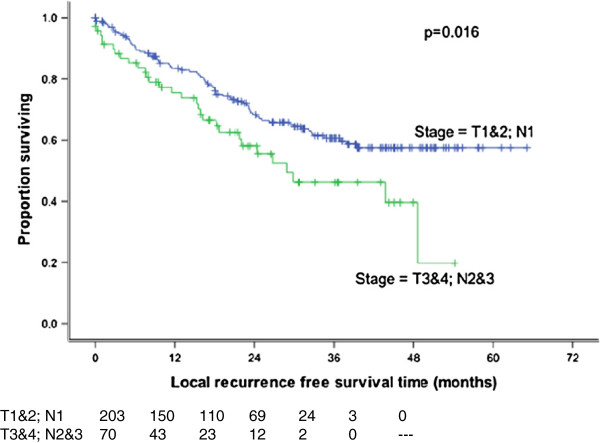
**Local recurrence free survival by stage.** Statistically significant benefit in local recurrence free survival for patient with early disease (p = 0.016).

Ozyar et al. [17] multivariate analysis showed that advanced nodal status was a strong significant unfavourable factor for DFS. Sham et al. [20] confirmed that nodal stage was prognostically significant even among patient groups stratified by size and degree of fixation of the neck nodes involved. Furthermore, in the present study, patients with more advanced disease (T3-4, N2-3) were also found to have a worse 3-year overall survival (p = 0.024) (Figure 5), disease free survival (p = 0.018) and local relapse free survival (p = 0.016) (Figure 6).

**Figure 7:**
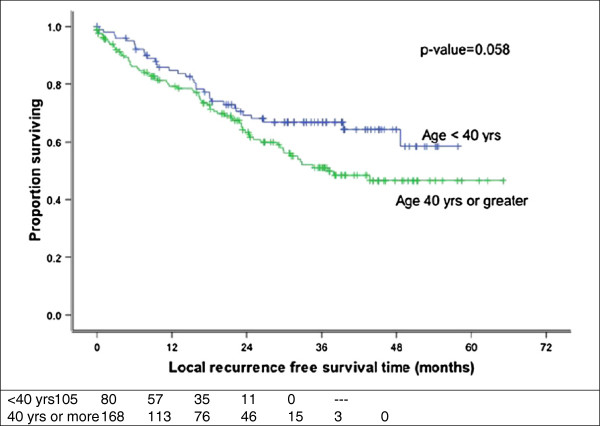
**Local recurrence free survival by age.** Improved RFS for patients younger than 40 years (p = 0.058).

**Figure 8:**
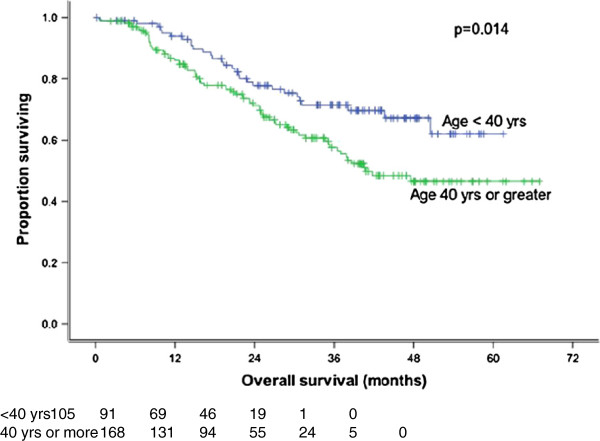
**Overall survival by age.** There is an advantage in overall survival for patients younger than 40 years (p = 0.014).

Sham and Choy [20] evaluated the records of 759 Stage I to IV nasopharyngeal carcinoma patients and found that the 5 years survival of patients less than 40 years old was better than those who were older. In the present study, age (<40 years versus > 40 years) significantly affected LRFS (p = 0.058) (Figure 7) and OS (p = 0.014) (Figure 8) at 3 years.

A meta-analysis of 8 trials by Baujat et al. [21] confirmed a survival benefit for cisplatin-based chemotherapy, a result similar to the Intergroup Study 0099 protocol [5] which showed chemoradiation to be superior for advanced nasopharyngeal carcinoma with respect to progression free survival and overall survival. The study by the Intergroup 0099 was the first to document a significant survival benefit for chemoradiaton versus radiotherapy alone. This has been confirmed by subsequent trials. As of today, it is not clear whether the adjuvant chemotherapy component of the Intergroup regimen contributed to its survival benefit. It is to be noted that the present study differed from the Intergroup study in that patients received neoadjuvant chemotherapy and concomitant chemoradiation but not adjuvant chemotherapy.

Cisplatin-based neoadjuvant or induction chemotherapy induces dramatic initial responses and good tolerability. Initial response rate is in the order of 93% with a 20% complete remission rate followed by 86% complete remission rate after radiotherapy [22]. One recent randomized phase II trial [23] demonstrated an overall survival benefit with cisplatin-docetaxel when compared to chemoradiotherapy alone. Two prospective clinical trials are currently underway. Other studies however, failed to demonstrate any benefit [24,25] for the neoadjuvant strategy.

In this study, 155 patients (57%) initiated treatment with the cisplatin-doxorubicin combination and 118 patients (43%) with cisplatin-5FU combination, due to the local treatment policy in one centre. Patient characteristics were equally distributed between the groups who received cisplatin/doxorubicin versus those receiving cisplatin/5FU. With regard to treatment arm, there were 5% more patients from the brachytherapy arm who completed the planned chemotherapy, which could potentially enhance results for the brachytherapy arm.

In a retrospective analysis of 145 patients, Leung et al. [18] reported that the 5 year major-complication-free survival rate was 89.5% for the brachytherapy group and 85.6% for the control group (p = 0.23). Similar to our study, there were no differences in major toxicity between patients receiving external radiation alone and patients receiving intracavitary brachytherapy where the following were evaluated: skin (p = 0.277), subcutaneous tissue (p = 1.0), mucous membranes (p = 0.209), salivary glands (p = 0.806), brain (p = 1.0), and trismus (p = 0.687). Patients commonly experienced low grade side effects such as mucous membrane mucositis and xerostomia. Conversely, high grade toxicity was not commonly observed with the exception of severe trismus in 15 patients in the control and 12 patients in the brachytherapy arm. It is important to note that longer follow-up may be required to observe late complications as nasopharyngeal ulceration and cranial nerve palsy as were reported by Teo et al. [16] where the median follow up were 49.4 months and 38.5 months for these two complications, respectively.

Levendag et al. [26] performed a pooled analysis including the patients in this study (n = 274) with two additional cohorts of patients with NPC treated in Rotterdam (n = 94) and Amsterdam (n = 89). The pool comprised a total of 457 patients. Thirty three patients were excluded because of having early (T1-T2 N0) disease. No significant difference in local recurrences was observed for the T3-T4 N + tumours. However, for T1-T2 N + tumours, significant differences in local control were found between patients treated with or without a brachytherapy boost in the pooled analysis, thus confirming previous studies for patients with early local disease.

This study is an example of the possibility of performing clinical trials in an international setting in developing countries on a common disease such as nasopharyngeal carcinoma. The overall results in terms of local tumour control, loco-regional control and survival appear lower than those in series from China using advanced conformal radiotherapy techniques. Notwithstanding the fact that this was a selected poor risk population of patients with advanced loco-regional disease, the reasons for this difference remain to be explored.

In conclusion, a clinical advantage of adding a brachytherapy boost in the setting of loco-regionally advanced nasopharyngeal carcinoma treated with cisplatin-based induction chemotherapy and combined chemo radiotherapy has not been demonstrated in this study.

## Competing interests

The authors declare having no conflicts of interests.

## Authors’ contributions

BV, PL and MEG developed the initial study design. IE performed statistical analysis. MEG, MA, NB, SA, YC accrued and treated patients. ER, MAW, JMB, BV, MEG and PL conducted interpretation of the study results and drafted the manuscript. All authors read and approved the final manuscript.
